# Supplementary material for: A multi-omics integrative analysis based on CRISPR screens re-defines the pluripotency regulatory network in ESCs
Source: Commun Biol. 2023 Apr 14;6:410. doi: 10.1038/s42003-023-04700-w (PMC10104827; doi:10.1038/s42003-023-04700-w)
Supplement: Supplementary file 2 — Supplementary Material [file 42003_2023_4700_MOESM2_ESM.pdf]

A multi-omics integrative analysis based on CRISPR screens re-defines the pluripotency regulatory network in ESCs

Yan Ruan[1,#], Jiaqi Wang[1,2,#], Meng Yu[1,3,#], Fengsheng Wang[1], Jiangjun Wang[1,4], Yixiao Xu[1], Lianlian Liu[1], Yuda Cheng[1], Ran Yang[1,5], Chen Zhang[1], Yi Yang[6], JiaLi Wang[1], Wei Wu[7], Guangxing Chen[3], Yi Huang[8], Yanping Tian[1,*], Junlei Zhang[1,*] & Rui Jian[1,*]

[1]Laboratory of Stem Cell & Developmental Biology, Department of Histology and Embryology, College of Basic Medical Sciences, Army Medical University, Chongqing, 400038, China.

[2]Institute of Immunology PLA & Department of Immunology, College of Basic Medical Sciences, Army Medical University, Chongqing, 400038, China.

[3]Department of Joint Surgery, The First Affiliated Hospital, Army Medical University, Chongqing, 400038, China.

[4]Department of Cell Biology, College of Basic Medical Sciences, Army Medical University, Chongqing, 400038, China.

[5]Department of Pathophysiology, College of High Altitude Military Medicine, Army Medical University, Chongqing, 400038, China.

[6]Experimental Center of Basic Medicine, College of Basic Medical Sciences, Army Medical University, Chongqing, 400038, China.

[7]Thoracic Surgery Department, Southwest Hospital, The First Affiliated Hospital, Army Medical University, Chongqing, 400038, China.

[8]Biomedical Analysis Center, Army Medical University, Chongqing, 400038, China.

[#]These authors contributed equally to this work.

*Corresponding author. Email: jianruilq2@aliyun.com (R.J.); zhangjunlei@aliyun.com (J.L.Z); tianyp1981@163.com (Y.P.T)

Keywords: multi-omics analysis, CRISPR screen, pluripotency regulatory network, ESC, cancer

List of supplementary materials

1. **Supplemental figures and legends**

Supplementary Figure 1, related to Figure 1.

Supplementary Figure 2, related to Figure 2.

Supplementary Figure 3, related to Figure 3.

Supplementary Figure 4, related to Figure 5.

Supplementary Figure 5, related to Figure 6.

Supplementary Figure 6, related to Figure 6.

Supplementary Figure 7, related to Figure 6.

Supplementary Figure 8, related to Figure 8.

2. **Supplemental tables**

Supplementary Table 1. Summary of the five screening studies.

Supplementary Table 2. A list of sgRNAs used in this work.

Supplementary Table 3. A list of shRNAs used in this work.

Supplementary Table 4. A list of primers used for qRT-PCR in this work.

Supplementary Table 5. A list of primers used for ChIP qRT-PCR in this work.

3. **Supplemental Data**

Supplementary Data 1. The lists of negative and positive selection genes of current screen. The GO and pathway terms enriched in negative selection genes of current screening.

Supplementary Data 2. The raw data of five screening studies.

Supplementary Data 3. The normalized results of five screens.

Supplementary Data 4. The lists of “ribosome”, “core TFs”, “low expression”, “non-essential”, “Top

1000” and “Top 100” genes.

Supplementary Data 5. The lists of “Differentiation”, “Low in mESC”, “High in mESC”, “Common (overlapped)” and “Context-specific” genes.

Supplementary Data 6. The list of iSRGS genes and pathway terms enriched in iSRGS.

Supplementary Data 7. ChIP-seq data of transcriptional regulators and histone markers.

Supplementary Data 8. HDBS of nine sub-classes.

Supplementary Data 9. Constituents of the functional modules.

Supplementary Data 10. GO terms enriched in module genes.

Supplemental figures and legends

Jian_Supplementary Figure 1

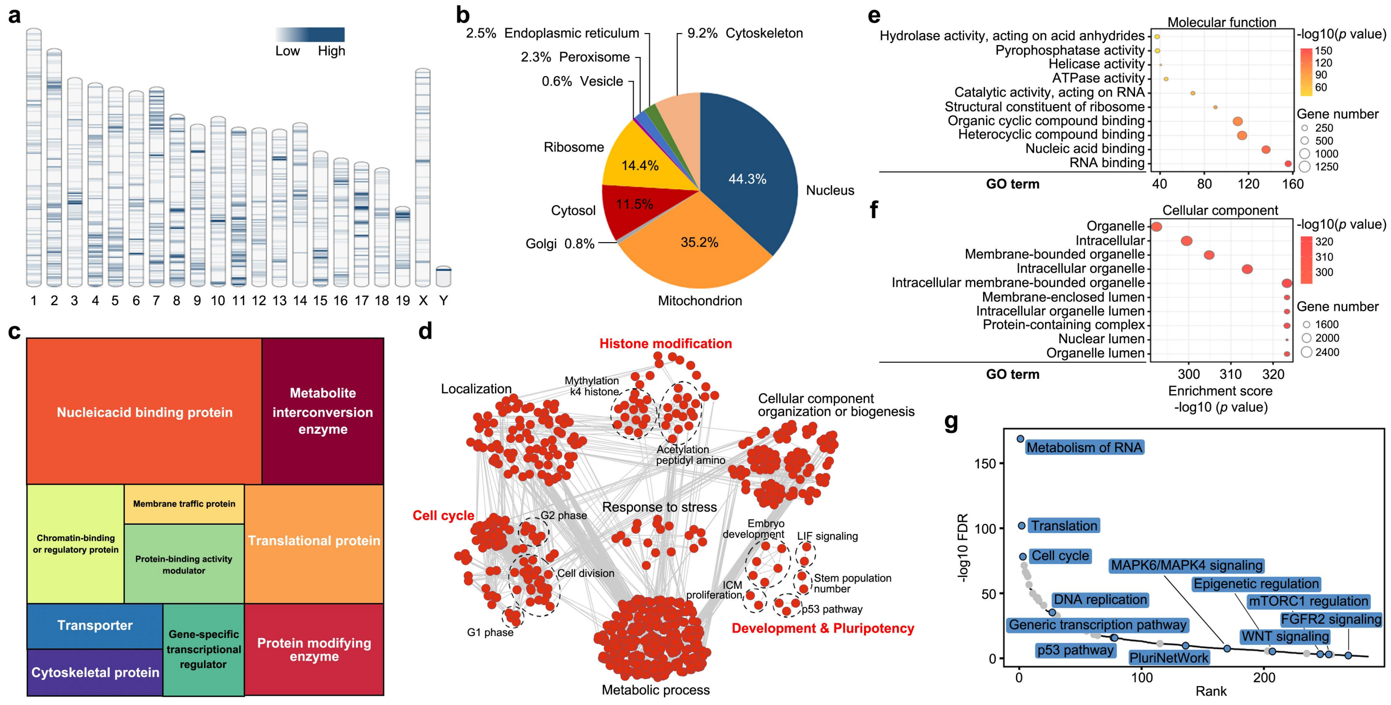

Figure 1_S. Characterization of the negative selection genes.

(a) Chromosomal distribution of genes. The colour of the line corresponds to the number of negative selection genes located in this region (blue is high, and grey is low).

(b) Distribution of the genes across cellular compartments.

(c) A tree map of the enriched protein functional categories. The square size is proportional to the percentage of genes involved in each functional category.

(d) Enrichment map networks of GO terms corresponding to the negative selection genes. Gene ontology was analysed by g:profiler and visualized by the Cytoscape plug-in: Enrichment Map. Nodes represent GO terms, node size represents the gene set size, and edges connect nodes that share common genes. Labels summarize the functions of gene set clusters.

(e, f) Molecular function (e) and cellular components (f) enriched in the negative selection genes. The top 10 enrichment terms are presented.

(g) Pathways enriched in the negative selection genes are ranked by enrichment significance (-$\log_{10}$ FDR). Blue dots indicate the pathways involved in pluripotency maintenance.

Jian_Supplementary Figure 2

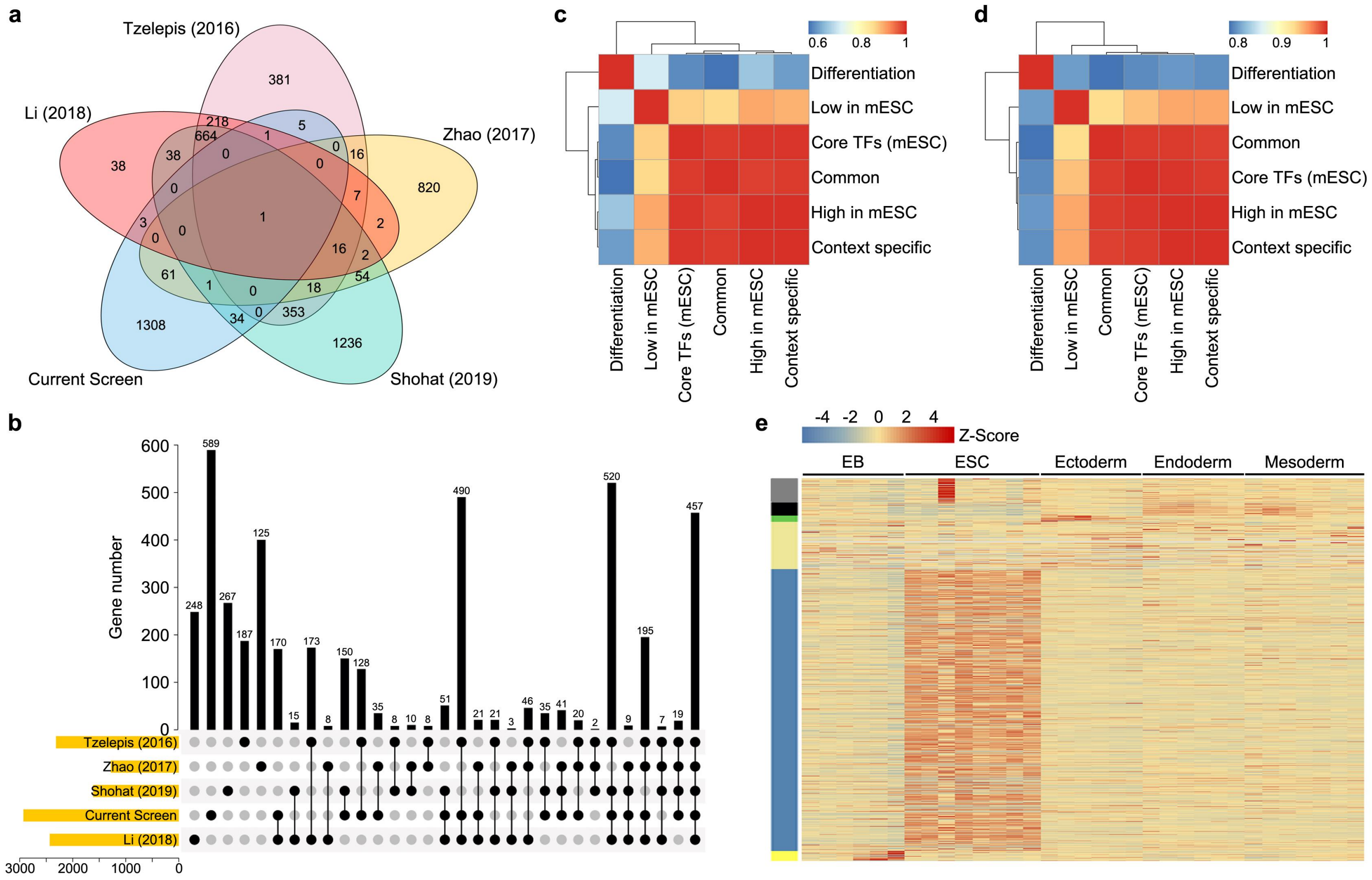

Figure 2_S. The expression profile of the common and context-specific gene sets.

(a) Venn diagram depicting a comparison of the negative selection results from different studies.

(b) The number of genes identified in all five screens, any four of the five screens, any three of the five screens, any two of the five screens and a single screen. The length of the yellow bar corresponds to the gene number of the indicated screening result.

(c, d) Comparison of the expression profiles of “Differentiation”, “Low in mESC”, “Core TFs (mESCs)”, “Common (overlapped)”, “High in mESC”, and “Context-specific” genes in Ep4.5d inner cell mass (c) and IB10 mESCs (d) (Supplementary Data 5) .

(e) The expression profiles of module genes in mESCs (ESC), EBs and directionally differentiated cells (ectoderm, endoderm and mesoderm). Colours in the left longitudinal bar represent the modules assigned by WGCNA. The cell colour intensity in the heatmap is proportional to the Z-Score (red is high, and blue is low).

Jian_Supplementary Figure 3

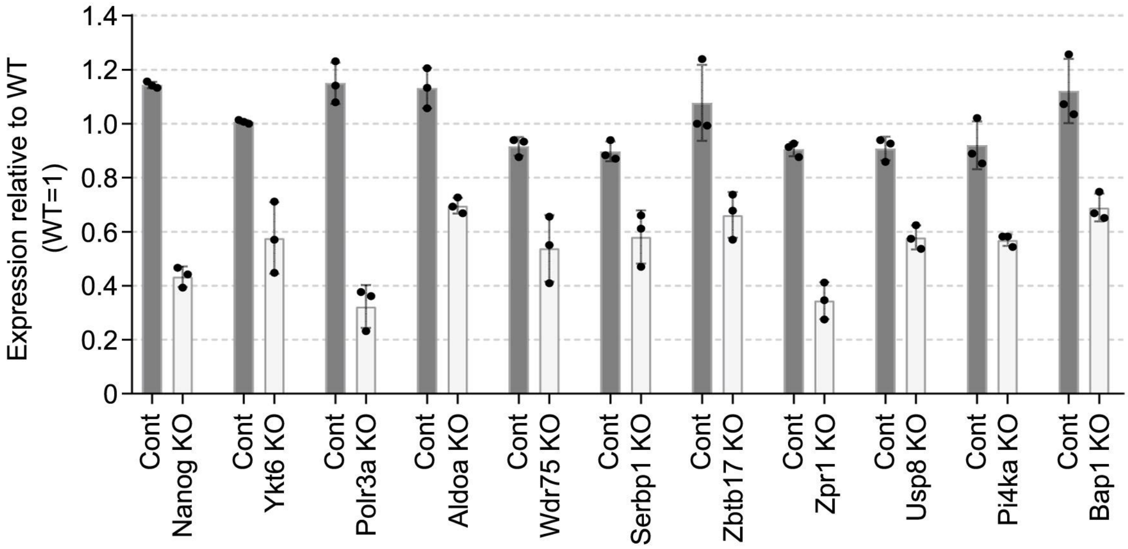

Figure 3_S. The silencing level of the candidate genes.

qRT-PCR analysis of the gene expression levels in cells that were infected with lentivirus carrying either control sgRNA or sgRNA targeting the indicated gene. All data is normalized to Gapdh and shown relative to WT ESCs (set at 1.0). Data are represented as mean ± SD; n = 3.

Jian_Supplementary Figure 4

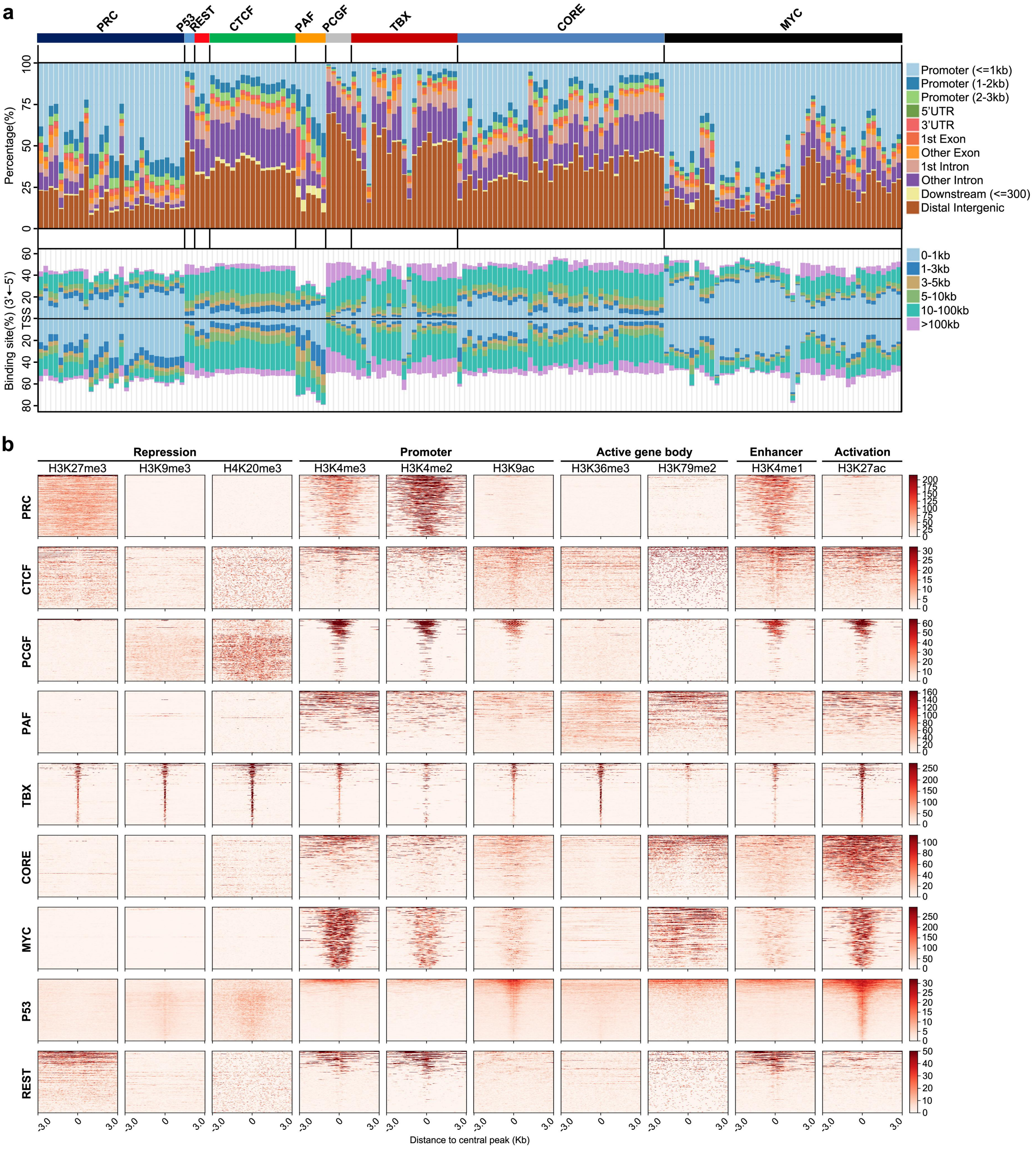

Figure 4_S. Genomic distributions and histone modifications of the sub-class binding sites.

(a) Genomic annotation (upper) and distance distribution (below) of the sub-class binding sites from TSSs.

(b) The histone modification signatures of the sub-classes. The reads per genome coverage (RPGC) of ten histone marks on nine sub-class binding sites are shown (see Methods section). In a single heatmap, each row (y

axis) indicates a specific co-occupancy site of the sub-class genes. Spots in the row indicate the histone modifications within 3 kb regions of the central binding peak (x axis). The spot colour corresponds to the normalized read count of the histone mark.

Jian_Supplementary Figure 5

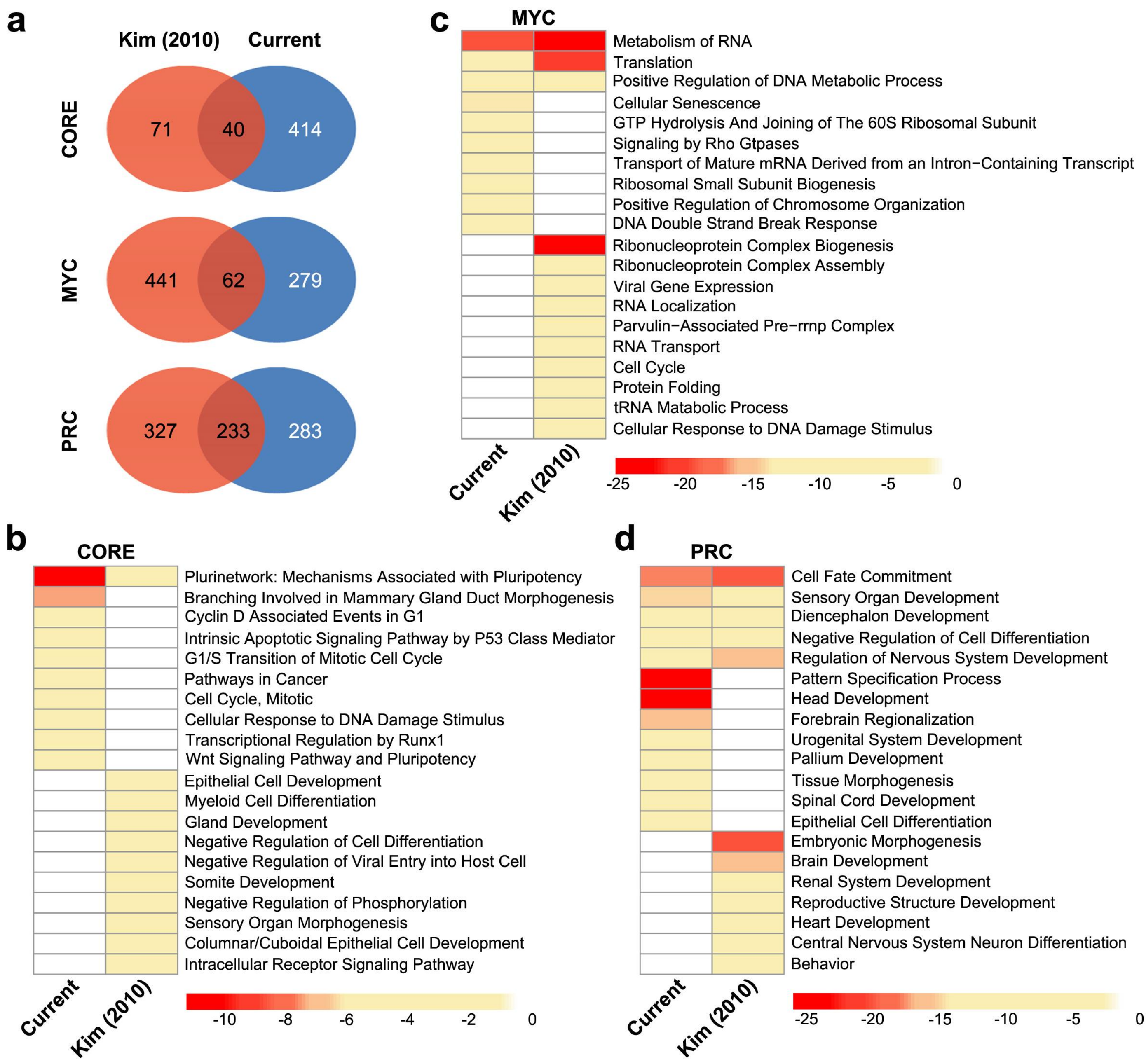

Figure 5_S. Comparison of the modules defined in previous and current studies.

(a) Comparison of the target genes of the CORE, MYC and PRC modules identified in Kim's and this study.

(b-d) Functional enrichment analyses identify the GO terms and pathways in two gene sets of the CORE (b), MYC (c) and PRC (d) modules. The high enrichment terms in each gene set are shown. The cell colour corresponds to $-\log_2$ (*p*-value).

Jian_Supplementary Figure 6

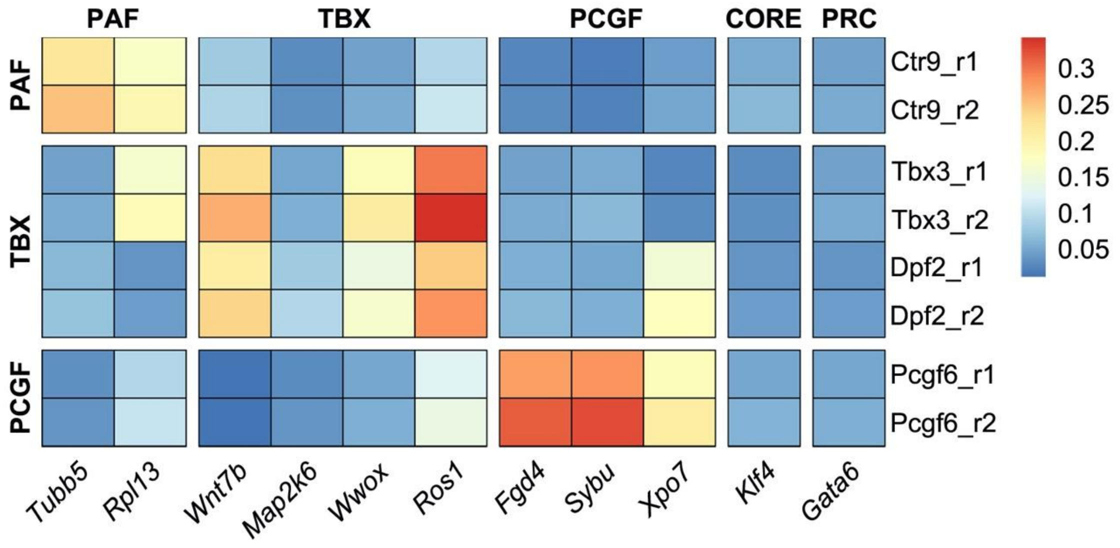

Figure 6_S. The validation of the targets occupied by PAF, TBX3 and PCGF module respectively.

ChIP assays were performed using anti-Flag antibody in Flag-tagged Ctr9 (for PAF module), Tbx3 (for TBX module), Dpf2 (for TBX module) and Pcgf6 (for PCGF module) transfected mESCs. Then 11 target genes in indicated modules were chosen for qPCR analysis. Values are expressed as a percent of input DNA. The cell colour intensity in the heatmap is proportional to the value.

Jian_Supplementary Figure 7

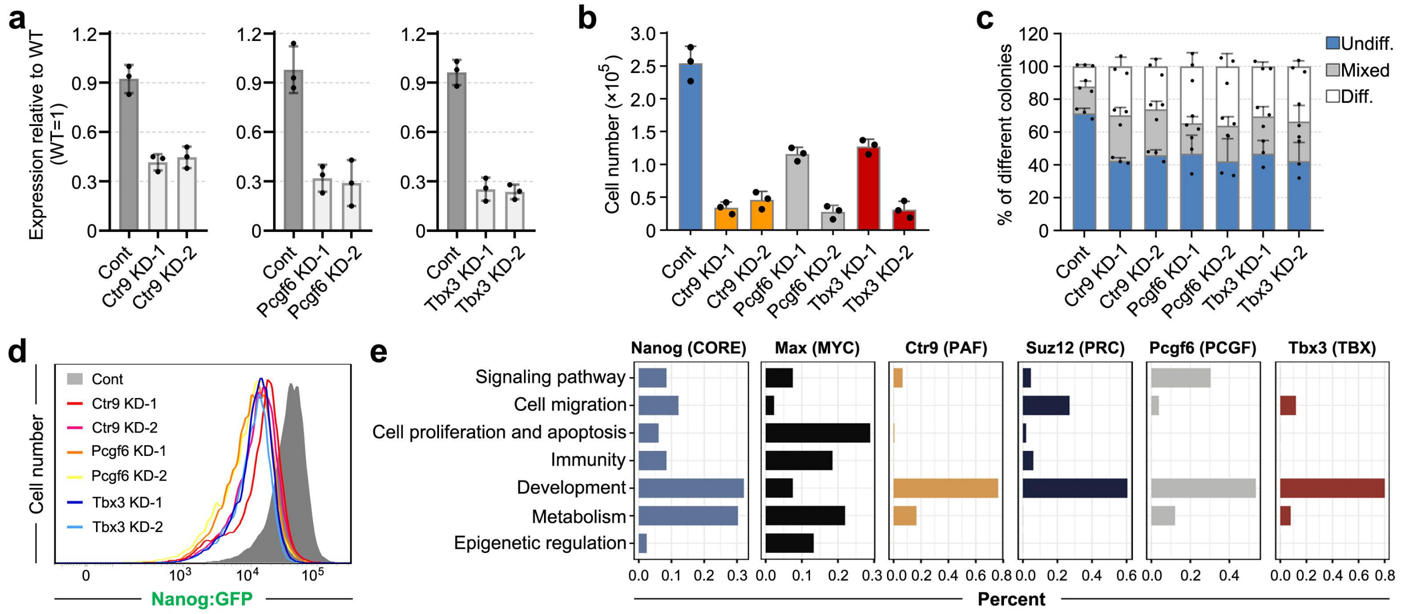

Figure 7_S. The functional analysis of PAF, TBX3 and PCGF modules in mESC.

(a) qRT-PCR analysis of the indicated gene expression in control (Cont) and knockdown (KD) cell lines. The major transcriptional factors of each module, Ctr9 (PAF), Pcgf6 (PCGF) and Tbx3 (TBX), were chosen for functional analysis. mESCs (Nanog-GFP reporter) were infected with lentivirus carrying control or shRNAs targeting the indicated gene. All data is normalized to Gapdh and shown relative to WT ESCs (set at 1.0).

(b) The indicated cells (1000 cells per cm^2 in 12-well plates) were cultured for four days with LIF and cell numbers were counted.

(c) Quantitative analysis of the colony formation assay in the indicated cell lines. Cells were plated at a clonal density and cultured with or without LIF for six days. Colonies were fixed, stained for AP, and scored as undifferentiated, mixed, or differentiated.

(d) FACS analysis for Nanog:GFP in the indicated lines.

(e) GO analysis for DEGs in ESCs knocking down the indicated gene. GO terms were categorized into seven classes based on their function. The scale bar shows the percentage of terms involved in each class. All experiments were repeated three times, and a representative result was shown. Data in a-c are represented as mean ± SD; n = 3.

Jian_Supplementary Figure 8

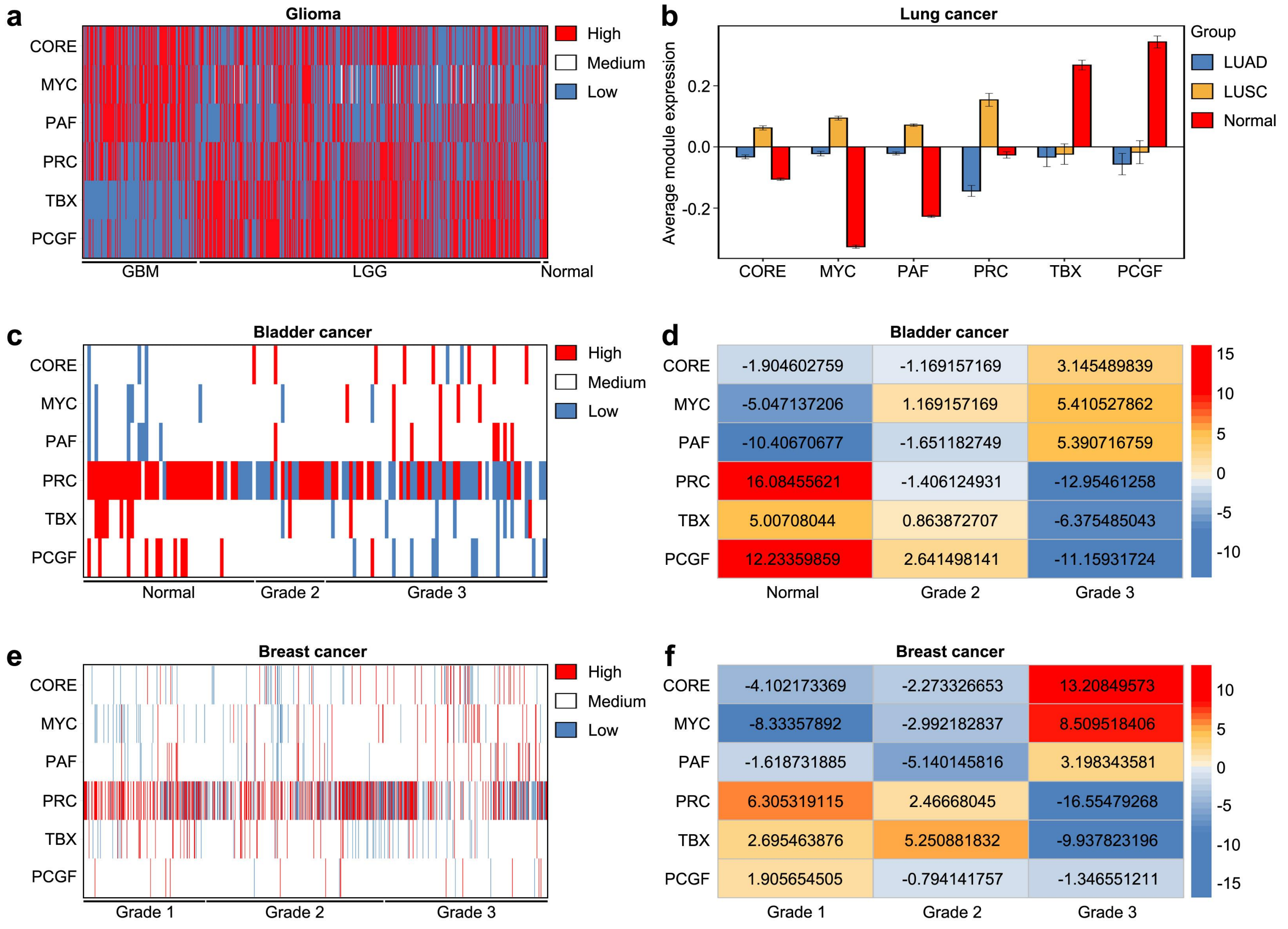

Figure 8_S. Module activity in various cancers.

(a) Enrichment pattern of each module gene (rows) across 1148 samples including 6 normal, 547 LGG and 595 GBM samples. Columns represent individual samples. Red indicates high-expression enrichment, blue indicates low-expression enrichment, and white indicates no significant enrichment.

(b) Average gene expression values (log_2) of each module in normal, LUAD and LUSC samples. Data are represented as the mean ± SEM. LUAD, lung adenocarcinoma. LUSC, lung squamous cell carcinoma.

(c, d) Module activity in bladder cancer (total 157 samples). Enrichment pattern of module genes in individual samples (c) and sample groups (normal: 48 samples, Grade 1: 19 samples and Grade 2: 90 samples) (d).

(e, f) Module activity in breast cancer (total 1097 samples). Enrichment pattern of module genes in individual samples (e) and sample groups (Grade 1: 217 samples, Grade 2: 393 samples and Grade 3: 487 samples) (f).

Supplemental tables

Supplementary Table 1. Summary of the five screening studies

Screen	Library	Cell line	Culture condition	Sample collection	Data analysis	Threshold
**Zhao et al., 2017**	GeCKO-v2 lenti-CRISPR virus library A (Addgene #1000000052)	mESC(TGET)	ES cells were maintained on MEF in DMEM supplemented with 15% FBS, 100 U ml^{-1} LIF, 0.1 mM Non-Essential Amino Acids Solution, 1× Glutamax , and 4.5 × 10^{-4} M MTG.	2 weeks post infection	HiTSelect	TOP1000
**Shohat et al., 2019**	Brie (Addgene, #73633)	mESC	DMEM supplemented with 15% FBS, 0.1 mM non-essential amino acids, 100 μM β-mercaptoethanol, 100 U ml^{-1} LIF, 2 mM L-glutamine, 3μM CHIR99021 and 1 μM PD0325901.	15 days post transfection	Simulation-based approach which based on comparing the fold change of gRNAs targeting each gene to the fold change of randomly selected control gRNAs	FDR<0.05
**Tzelepis et al., 2016**	Mouse genome-wide CRISPR guide RNA library V2 (Addgene, #67988)	mESC (JM8)	DMEM supplemented with 100 μM β-mercaptoethanol, 2 mM L-glutamine, 100 U ml^{-1} LIF and 15% FBS.	14 days post transfection	MAGeCK	FDR<0.1/Genes that had 3 or more gRNAs with ≥ 4-fold reduction
**Li et al., 2018**	Mouse genome-wide CRISPR guide RNA library V2 (Addgene, #67988)	mESC (REX-GFP; Wray et al., 2011)	ES cells were cultured on feeder cells in KO-DMEM supplemented with 15% FBS, 1% GlutaMAX, 1% NEAA, 0.1 mM β-mercaptoethanol, and 1000 U/ml LIF.	15 days post transfection	MAGeCK	FDR<0.1
**Current screen**	Brie (Addgene, #73633)	mESC(R1)	DMEM supplemented with 100 μM β-mercaptoethanol, 2 mM L-glutamine, 1000 U ml^{-1} LIF, 15%	18 days post transfection	MAGeCK	$p<0.05$

KSR and 5% FBS.

Supplementary Table 2. Sequences of sgRNAs against target genes.

Target Gene	sgRNA (No.1)	sgRNA (No.2)
non-target	GACCAACCTTACGGTAACTC	
mNanog	AGAACTATTCTTGCTTACAA	TCAAGTCCTGAGGCTGACAA
mYkt6	CTTTACAAGTCAACTGATTG	CCGGCTACAATCCAGTACAC
mPolr3a	AACCACGCCCCCTTGTTGTA	GGCTGTCATTGGTATCTTAC
mAldoa	CCTTGCCCGGAGCCACAATG	TGACATCGCTCACCGCATTG
mWdr75	GAGTGTGTGCACATTCTGCA	ACGGAATCTTCCGCGTGGAG
mSerbp1	AAGACCCGATCAACAACTAC	CTGAAGCATGAGGACAAACG
mZbtb17	GAGCCTTAGCCCTGAGAACG	AGAACATCGTCCACGTTCTC
mZpr1	GTTCCAGCCACAGTGTTCGC	CCACTTCTCTGTTCATGTCC
mUsp8	ATGCAGACTAGATCGTGATG	TGCGGCAGCCAAACGCTCTG
mPi4ka	GCAAGCCACATCAGACAGCA	ATGGGATCGCATACTTGCAA
mBap1	TCAAATGGATCGAAGAGCGC	GCCCACGCTGAGCCGAATGA

Supplementary Table 3. Sequences of shRNAs against target genes.

Target Gene	shRNA (No.1)	shRNA (No.2)
control	GACCAACCTTACGGTAACT	
m/hCtr9	GAGCCTATACTATTGATCC	GAAGAAGTTTGAGAGGATA
mPcgf6	GAAGGTCCTAGAGTCAGTG	GACATAGTATACAAATTAG
mTbx3	GTCGTCACTTTCCACAAAC	GAATGACAAGATAACTCAG
hNANOG	GCAACCAGACCTGGAACAA	GATGAGTGAAACTGATATTAC
hMYC	GATGAGGAAGAAATTGATG	CAGTTGAAACACAAACTTGAA

Supplementary Table 4. Primers for qRT-PCR analysis.

Gene	Primer Sequence (5’ - 3’)	
	Forward	Reverse
GAPDH	AGGTCGGTGTGAACGGATTTG	TGTAGACCATGTAGTTGAGGTCA
mNanog	TCTTCCTGGTCCCCACAGTTT	GCAAGAATAGTTCTCGGGATGAA
mYkt6	GGCGCCATGAAGCTGTACAG	TGACCACACCTGCCAGACTG
mPolr3a	CCATGGTGAAGGAGCAGTTC	GGTAGCTCGAGATCAATGTAG
mAldoa	CACCCAGCAACAGACAGAGT	ATGCAGGGATTCACACGGTC
mWdr75	GTTCTGTACTTCTCACTCTGA	CAGGTGACCAGGCTTTCCAT
mSerbp1	AGTTGCGTAAAGAGTCCCAG	GATAGGCCGTTCGATAATCG
mZbtb17	GCTGGAGTTCATGTACACTG	TTGTCTCCTCCTTCCACAGC
mZpr1	TGCTCAAGCCCTCGTCCATG	GAAGGTCAAGGAGCACAGTAG
mUsp8	TCCTAAAGAACTCTACCTCAG	AGTTTAAGGCTTTCGGAGAGT
mPi4ka	AGACGGAGAGATGCAGTGAT	TATCAGGCATGGGATCGCAT
mBap1	AGATGAATAAGGGCTGGCTG	GCTGGTGAGCAAAGAACATG
mCtr9	ACAATGAGTCTGTGCAGTCA	AACCTAGTCGCTATCATCTG
mPcgf6	TGCTAATGAAGACACCGGAC	TGAAGAACAAGCAGACCGTC
mTbx3	GCCAGTGCACTTTGTTAGATG	TGGTATCTAGCATAGTCTCTG
hCtr9	GATCAGGACTCAGACAGTGA	TGTTCTGAGCCTCTATCTGA
hMYC	AACACAAACTTGAACAGCTACG	GTTGTGAGGTTGCATTTGATCA
hNanog	ACCTGAAGACGTGTGAAGATGA	AACACGTGGTTTCCAAACAAGA

Supplementary Table 5. Primers for ChIP qRT-PCR analysis.

Target	Primer Sequence (5' - 3')	
	Forward	Reverse
mTubb5	CATCGCTTATCACCTCCCAG	GCGTGTCTGGAATTGAAATGA
mRpl13	CAGTGGGAGGTTAGAGCGTA	CCCAACACTAACAGACACTG
mWnt7b	CTCTCTATACCTCTTTACCAG	GACAAGGATTCCAGGCAAAG
mMap2k6	GAGGAGGCAGTCACAAATGA	CATGAAAGGAAAGCAGAGTTC
mWwox	GGTTGAAGCCTGGCACATCT	TGAAAATCTTCAGGAATGGTGA
mRos1	ATACACACACAGCTGCCACT	AACTGAGTTCTGGCTACCTG
mFgd4	CAAGACCACTCACCATCATC	TCCTTCTTGATGTCCTCTAC
mSybu	ATCTCAGAACCGCTCCATGA	CTGTTTATCTGGAGCTCTGC
mXpo7	CACCATCTTCAGCTCCAGAC	GTTGTCTGGAGTCGAAGATG
mKlf4	CACTAGGGTCCCATTGCAGC	AACTCGCTCCCATCAACTTAG
mGata6	GTGACTAAATTGGATGAGAAC	GAATTCAGGAAGTTGGCGAGA
