# Supplementary material for: A multi-omics integrative analysis based on CRISPR screens re-defines the pluripotency regulatory network in ESCs
Source: Commun Biol. 2023 Apr 14;6:410. doi: 10.1038/s42003-023-04700-w (PMC10104827; doi:10.1038/s42003-023-04700-w)
Supplement: Supplementary file 3 — Description of Additional Supplementary Files [file 42003_2023_4700_MOESM3_ESM.pdf]

Description of Additional Supplementary Files

File name: Supplementary Data 1.
Description: The lists of negative and positive selection genes of current screen.TheGO and pathway terms enriched in negative selection genes of current screening.

File name: Supplementary Data 2.
Description: The raw data of five screening studies.

File name: Supplementary Data 3.
Description: The normalized results of five screens.

File name: Supplementary Data 4.
Description: The lists of “ribosome”, “core TFs”, “low expression”, “non-essential”, “Top 3 1000” and “Top 100” genes.

File name: Supplementary Data 5.
Description: The lists of “Differentiation”, “Low in mESC”, “High in mESC”, “Common(overlapped)” and “Context-specific” genes.

File name: Supplementary Data 6.
Description: The list of iSRGS genes and pathway terms enriched in iSRGS.

File name: Supplementary Data 7.
Description: ChIP-seq data of transcriptional regulators and histone markers.

File name: Supplementary Data 8.
Description: HDBS of nine sub-classes. Supplementary Data 9. Constituents of the functional modules.

File name: Supplementary Data 10.
Description: GO terms enriched in module genes.
