# Supplementary material for: A multi-omics integrative analysis based on CRISPR screens re-defines the pluripotency regulatory network in ESCs
Source: Commun Biol. 2023 Apr 14;6:410. doi: 10.1038/s42003-023-04700-w (PMC10104827; doi:10.1038/s42003-023-04700-w)
Supplement: Supplementary file 5 — Reporting Summary [file 42003_2023_4700_MOESM5_ESM.pdf]

nature portfolio

Corresponding author(s): Rui Jian

Last updated by author(s): Nov 30, 2022

Reporting Summary

Nature Portfolio wishes to improve the reproducibility of the work that we publish. This form provides structure for consistency and transparency in reporting. For further information on Nature Portfolio policies, see our Editorial Policies and the Editorial Policy Checklist.

Statistics

For all statistical analyses, confirm that the following items are present in the figure legend, table legend, main text, or Methods section.

n/a	Confirmed	
☐	☒	The exact sample size (*n*) for each experimental group/condition, given as a discrete number and unit of measurement
☐	☒	A statement on whether measurements were taken from distinct samples or whether the same sample was measured repeatedly
☐	☒	The statistical test(s) used AND whether they are one- or two-sided<br>*Only common tests should be described solely by name; describe more complex techniques in the Methods section.*
☒	☐	A description of all covariates tested
☐	☒	A description of any assumptions or corrections, such as tests of normality and adjustment for multiple comparisons
☐	☒	A full description of the statistical parameters including central tendency (e.g. means) or other basic estimates (e.g. regression coefficient) AND variation (e.g. standard deviation) or associated estimates of uncertainty (e.g. confidence intervals)
☐	☒	For null hypothesis testing, the test statistic (e.g. *F*, *t*, *r*) with confidence intervals, effect sizes, degrees of freedom and *P* value noted<br>*Give P values as exact values whenever suitable.*
☒	☐	For Bayesian analysis, information on the choice of priors and Markov chain Monte Carlo settings
☐	☒	For hierarchical and complex designs, identification of the appropriate level for tests and full reporting of outcomes
☐	☒	Estimates of effect sizes (e.g. Cohen's *d*, Pearson's *r*), indicating how they were calculated

Our web collection on statistics for biologists contains articles on many of the points above.

Software and code

Policy information about availability of computer code

Data collection	*Provide a description of all commercial, open source and custom code used to collect the data in this study, specifying the version used OR state that no software was used.*
Data analysis	programs used:<br>MAGeCK 0.5.9.3<br>R 4.1.1<br>GSEA 4.1.0<br>Cytoscape 3.8.2<br>RIdeogram(R package)<br>WGCNA 1.7-03(R package)<br>SRA-Toolkit 2.9.2<br>FastQC 0.11.9<br>Trim_Galore 1.18<br>Bowtie 2.3.4.1<br>MACS 2.1.4<br>BEDTools 2.26.0<br>deepTools 3.1.3<br>ChIPseeker 1.28.3(R package)<br>AdaSampling 1.3(R package)<br>HISAT2 2.1.0<br>featureCounts 1.6.0

AUCell(R package)
survival 3.2-13(R package)

For manuscripts utilizing custom algorithms or software that are central to the research but not yet described in published literature, software must be made available to editors and reviewers. We strongly encourage code deposition in a community repository (e.g. GitHub). See the Nature Portfolio guidelines for submitting code & software for further information.

Data

Policy information about availability of data

All manuscripts must include a data availability statement. This statement should provide the following information, where applicable:

- Accession codes, unique identifiers, or web links for publicly available datasets
- A description of any restrictions on data availability
- For clinical datasets or third party data, please ensure that the statement adheres to our policy

The raw data of the four screening studies, Shohat (2019), Tzelepis (2016), Li (2018), and Zhao (2017), were downloaded from the supplementary material of these references. RNA-seq data were downloaded from GEO with accession number GSE120224 for EB differentiation of mESC (0d versus EB12d) (Figure 2e; Figure 7a); GSE143371 for EB differentiation of hESC (0d,5d,10d) (Figure 7d); and GSE44067 (Figure 1i; Figure 2c) and GSE53387 (Figure 2e) for mESCs cultured in LIF/serum. RNA-seq data were downloaded from EMBL-EBI with accession number E-MTAB-4904 for three germ layer directional differentiation of mESCs (Figure 2e; Figure 7b) and E-MTAB-2958 and E-MTAB-2959 for E2.5-E4.5 mouse embryos (Figure 7c). scRNA-seq data were downloaded from GEO with accession number GSE65525 for mESCs cultured with LIF/serum (Figure S2d); GSE116165 for mouse E4.5 embryos (Figure S2c) and GSE120963 for mouse embryo spatiotemporal scRNA-seq (E5.5-E7.5) (Figure 7c). Microarray data of mESC EB differentiation were downloaded from GSE3749 (Figure 4b). mESC histone ChIP-seq data were downloaded from GEO with accession number GSE11724 for H3K79me2; GSE12241 for H4K20me3; GSE24164 for H3K27ac; GSE25532 for H3K27me3; GSE27827 for H3K4me2; GSE29218 for H3K4me3; GSE29413 for H3K9me3; GSE30203 for H3K4me1; GSE31284 for H3K9ac; and GSE41589 for H3K36me3. RNA-seq data for lung cancer and gliomas as well as the clinical survival data of gliomas were downloaded from the Xena dataset (https://xenabrowser.net/datapages) (Figure 8a-c; Figure S5a-b). The breast and bladder normalized expression data files as well as sample annotations were found at http://jura.wi.mit.edu/bioc/benporath/ (Figure S5 c-f). The ChIP-seq data for transcriptional regulators were downloaded and are listed in Supplementary Data 7. There are not restrictions on data availability in this manuscript.

Human research participants

Policy information about studies involving human research participants and Sex and Gender in Research.

Reporting on sex and gender	The study did not involve human research participants
Population characteristics	-
Recruitment	-
Ethics oversight	-

Note that full information on the approval of the study protocol must also be provided in the manuscript.

Field-specific reporting

Please select the one below that is the best fit for your research. If you are not sure, read the appropriate sections before making your selection.

☒ Life sciences ☐ Behavioural & social sciences ☐ Ecological, evolutionary & environmental sciences

For a reference copy of the document with all sections, see nature.com/documents/nr-reporting-summary-flat.pdf

Life sciences study design

All studies must disclose on these points even when the disclosure is negative.

Sample size	No statistical method was used to determine the sample size. Sample size was chosen based on standards in the field. Samples size and number of independent experiments are stated in figure legends or in Methods section.
Data exclusions	No data was excluded from the analysis.
Replication	The screen experiments include two independent biological replicates. Other experiments were performed at least 3 times.
Randomization	No animals and/or human participants were used and no randomization was need to perform for the experimental groups.
Blinding	Investigators were not blinded to group allocation during data collection and/or analysis.

Reporting for specific materials, systems and methods

We require information from authors about some types of materials, experimental systems and methods used in many studies. Here, indicate whether each material, system or method listed is relevant to your study. If you are not sure if a list item applies to your research, read the appropriate section before selecting a response.

Materials & experimental systems

n/a	Involved in the study
☒	☐ Antibodies
☐	☒ Eukaryotic cell lines
☒	☐ Palaeontology and archaeology
☒	☐ Animals and other organisms
☒	☐ Clinical data
☒	☐ Dual use research of concern

Methods

n/a	Involved in the study
☒	☐ ChIP-seq
☒	☐ Flow cytometry
☒	☐ MRI-based neuroimaging

Eukaryotic cell lines

Policy information about cell lines and Sex and Gender in Research

Cell line source(s)	Mouse、 Human
Authentication	Cell lines were validated by their gene expression patterns and phenotypes.
Mycoplasma contamination	All cell lines were tested negative for mycoplasma contamination.
Commonly misidentified lines (See ICLAC register)	No commonly misidentified cell lines were used.
